# OPG and BAFF as predictive biomarkers of the severity of SARS‐CoV‐2 infection

**DOI:** 10.1111/jcmm.70189

**Published:** 2025-01-31

**Authors:** Andy Ruiz, Carlos Peña‐Bates, Lucero A. Ramon‐Luing, Daniel Baca‐Nuñez, Marco Antonio Vargas, Karen Medina‐Quero, Neptali Gutierrez, Joel A. Vázquez‐Pérez, Ramcés Falfán‐Valencia, Gloria Pérez‐Rubio, Carolina Di Benedetto, Ivette Buendia‐Roldan, Moisés Selman, Paola Betancur, Leslie Chavez‐Galan

**Affiliations:** ^1^ Research Unit Instituto Nacional de Enfermedades Respiratorias Ismael Cosío Villegas Mexico City Mexico; ^2^ Research Unit Escuela Militar de Graduados de Sanidad Mexico City Mexico; ^3^ Department of Radiation Oncology University of California San Francisco California USA

**Keywords:** BAFF, COVID‐19, OPG, severity, TRAIL

## Abstract

Molecules of the tumour necrosis factor superfamily (TNFSF) are key players in immune regulation; an increase in some TNFSF molecules has been reported during severe COVID‐19. In this study, we profiled and evaluated TNFSF members in the serum of COVID‐19 vaccine‐naïve patients to identify potential biomarkers associated with disease severity. Our data show that TRAIL serum levels are lower in severely affected patients than those mildly affected by COVID‐19 (AUC 0.8, *p* = 0.0003). On the contrary, OPG and BAFF serum levels are higher in severe COVID‐19 compared to mild COVID‐19 cases (AUC 0.8, *p* = 0.0001; AUC 0.7, *p* = 0.0012; respectively) and moderate COVID‐19 cases (OPG *p* < 0.01), BAFF (*p* < 0.05). At the transcriptional level, TRAIL, OPG and BAFF are elevated in severe compared to mild COVID‐19 cases, with OPG and BAFF also higher in moderate compared to mild COVID‐19 patients. Additionally, we found that APRIL, LIGHT, CD30L and CD40L protein‐levels are higher in COVID‐19 patients compared to healthy donors but not significantly different between various COVID‐19 clinical statuses. Finally, we found that TNF‐α, TNF‐β, RANKL and TWEAK protein levels were not affected during COVID‐19. Our work identifies OPG and BAFF as potential biomarkers and therapeutic targets for preventing severe COVID‐19. Due to the opposite contradictory levels of TRAIL (protein/transcriptional level), its role during COVID‐19 should be elucidated and clarified with more in‐depth studies.

## 1 INTRODUCTION

The international emergency of COVID‐19, caused by SARS‐CoV‐2, has already been declared an endemic disease. Despite a large part of the population being vaccinated, patients continue to present severe complications, mainly related to factors such as age, the presence of comorbidities (hypertension, diabetes), or chronic diseases such as asthma, coronary heart disease or chronic kidney disease.^1^, ^2^

The storm of cytokines is a critical immunologic feature during COVID‐19, leading to hyperinflammation, which has been directly linked to severe clinical characteristics.^3^ The tumour necrosis factor superfamily (TNFSF) members are molecules involved in these processes. It comprises 19 type II transmembrane proteins characterized by a conserved trimeric C‐terminal domain. They are synthesized as membrane‐bound proteins and released as soluble proteins after proteolytic processes. TNFSF members have been implicated in processes regulating innate and adaptive immune responses.^4^ As described below, some TNFSF members have been proposed as biomarkers in chronic, infectious, and autoimmune diseases.

The TNF‐related apoptosis‐inducing ligand (TRAIL) is a TNFSF member; it induces apoptosis through a death domain; it has a homotrimeric structure and is critical in immune regulation. TRAIL has five receptors, from TRAIL‐R1 to TRAIL‐R4 and osteoprotegerin (OPG). Apoptosis is initiated by TRAIL binding to TRAIL‐R1 (DR4) and TRAIL‐R2 (DR5/TRICK2/KILLER) receptors, known as death receptors, whereas TRAIL‐R3 (DcR1/TRID/LIT) and TRAIL‐R4 (DcR2/TRUNDD) receptors inhibit apoptosis because they lack functional death domains.^5^, ^6^ Although TRAIL has been extensively studied in the context of cancer, it has also been suggested that its low levels can be associated with COVID‐19 severity.^7^

The proliferation‐inducing ligand (APRIL) and the B‐lymphocyte activation and maturation promoter (BAFF) are TNFSF members; their presence is associated with B‐cell response. Reports indicate that APRIL/BAFF heteromers increase in autoimmune diseases, and both ligands are involved in T‐cell‐independent antibody production in patients with parasitic infections.^8^, ^9^, ^10^

OPG is involved in the regulation of bone remodelling. It circulates in the blood as a monomer or homodimer and is bound to other ligands such as TRAIL and receptor activator of NF‐κB ligand (RANKL). OPG is produced by various immune cells and endothelial cells. A high concentration of this ligand in the circulation is considered a high risk for vascular pathologies and bone mass deterioration in COVID‐19.^5^, ^11^ RANKL induces osteoclast formation, maturation of immune cells, development of secondary lymphoid organs and regulation of fever in infections.^12^ Moreover, soluble tumour necrosis factor receptor 1 (TNFR1) levels are increased in severe COVID‐19. Interestingly, not all severe cases have increased soluble TNF levels, but those with high TNF levels activate PANoptosis, a type of cell death associated with inflammation.^13^, ^14^

Some TNFSF members have been suggested as biomarkers in the COVID‐19 context; however, no study has comprehensively evaluated the distribution of diverse TNFSF members. This analysis is imperative to search for accurate biomarkers, considering that some members are receptors or ligands, and consequently, the level of one could be influenced by the presence of the other.

## 2 MATERIALS AND METHODS

### 2.1 Ethics statement

This study received ethical approval from the Institutional Ethics Committee of the Instituto Nacional de Enfermedades Respiratorias Ismael Cosío Villegas (protocol number B09‐22). It adhered to the principles outlined in the 1964 Helsinki Declaration. Patients or their families provided written informed consent following the ethical standards established by the Institutional Ethics Committee.

### 2.2 Study design, population, and blood sample collection

Patient recruitment occurred at the onset of the COVID‐19 pandemic at the Hospital Central Militar (HCM) and at the Instituto Nacional de Enfermedades Respiratorias Ismael Cosío Villegas (INER). Ninety‐eight COVID‐19 patients diagnosed through reverse transcription‐polymerase chain reaction (RT‐PCR) testing on nasopharyngeal swabs were enrolled in this study. Blood samples were collected between May and September 2020 during the first COVID‐19 wave, and a healthy donor group (HD, *n* = 30) was included. It is important to note that patients and HD were naïve to COVID‐19 vaccination at study time.

As reported in previous studies, COVID‐19 patients were categorized into three groups based on the severity of the disease: 32 were classified as mild, 29 as moderate and 37 as severe. Following WHO definitions, this classification was performed using the CARE score, the COVID‐19 Treatment Guidelines score, and the Radiographic Assessment of Lung Edema (RALE) from the National Institutes of Health. All COVID‐19 patients showed similar clinical inflammatory parameters, but mild patients used shorter hospitalization days and had less affected oxygen saturation.

### 2.3 Blood sample

Clinical laboratory staff obtained blood samples for clinical tests from COVID‐19 patients at hospital admission. HD samples were obtained from subjects with a negative COVID‐19 diagnosis. For both HD and COVID‐19 patients, a blood sample was collected in a tube with spray‐coated EDTA (BD Vacutainer 367863, Franklin Lakes, NJ, USA) to obtain peripheral blood mononuclear cells (PBMCs), followed by RNA and DNA extraction. A second blood sample was collected in a tube containing a clot activator and serum separator gel (BD Vacutainer 368159, Franklin Lakes, NJ, USA) to collect serum, which was kept at −20°C until use, avoiding freezing and thawing processes.

### 2.4 ELISA sandwich assays

TRAIL levels were evaluated in serum using an Enzyme‐Linked Immunosorbent Assay (ELISA). The Human TRAIL ELISA (Cat. No. BMS2004, ThermoFisher Scientific, Waltham, MA, USA) has a 5 pg/mL sensitivity. Quantification was performed following the manufacturer's instructions using a standard curve. The optical density (450 nm) was measured using a microplate reader (Imark, Bio‐Rad, Hercules, CA, USA).

### 2.5 LEGENDplexTM assay

Our study employed the LEGENDplex kit, a multiplex bead‐based assay panel (LEGENplexTM Human TNFSF, Cat. N. 741308, BioLegend, San Diego, CA). These panels were explicitly chosen for quantifying the protein concentrations of TNF superfamily ligands, including OPG, APRIL, TRAIL, sCD40L (Soluble CD40 Ligand), TWEAK (TNF‐like Weak Inducer of Apoptosis), LIGHT (TNFSF14, Ligand for Herpesvirus Entry Mediator), FasL (Fas Ligand), RANKL, TNF‐α (tumour necrosis factor alpha), TNF‐β (Tumour Necrosis Factor Beta), BAFF, CD30L (CD30 Ligand). The bead assays were conducted strictly following the manufacturer's guidelines. Data were acquired using flow cytometry (FACS Aria IIu, Becton Dickinson) and analysed with the LEGENDplex™ Data Analysis Software Suite.

### 2.6 RNA extraction and synthesis of cDNA

Peripheral blood specimens from HD and individuals confirmed to have SARS‐CoV‐2 infection were processed to extract total RNA. To ensure RNA stability, blood samples were preserved at −70°C using DNA/RNA Shield solution (Cat. No. R1100‐250, Zymo Research, Irvine, CA, USA). The samples were diluted at a ratio of 1:3 with the solution, thoroughly mixed, and subjected to room temperature incubation to facilitate cell lysis and then stored in a frozen state. Before RNA extraction, samples were thawed, and RNA was isolated using the RNeasy Micro Kit (Cat. No. 74106, Qiagen, Hilden, Germany), adhering to the manufacturer's protocol. Genomic DNA contamination was eliminated using the RNA‐Free DNAse Set (Cat. No. 79254, Qiagen, Hilden, Germany). RNA was then eluted in 30 μL of nuclease‐free water. RNA was quantified using the Qubit™ assay kit with the Qubit 2.0 Fluorometer (Life Technologies, Waltham, MA, USA). For the synthesis of first‐strand cDNA, 10 ng of total RNA was utilized, employing the high‐capacity cDNA reverse transcription kit (Cat. No. 4368814, Applied Biosystems, Waltham, MA, USA) in a total reaction volume of 20 μL, as per manufacturer's instructions.

### 2.7 Quantitative real‐time PCR in gene expression analysis

Quantitative real‐time PCR (qPCR) was used for gene expression analysis. The qPCR was performed using the StepOnePlusTM Real‐Time PCR Systems (applied biosystems) with SYBR Green. The specific forward and reverse primers reported in the literature for each target gene were *TNFRSF11B* (OPG), 5′‐GGCAACACAGCTCACAAGAA −3′ and 5’‐CGGTAAGCTTTCCATCAAGC‐3′; *TNFSF13B* (BAFF) 5′‐GAGAAGCTGCCAGCAGGA‐3′ and 5′‐GGAGCTGGTGGTTCAAAGATT‐3′; and *TNFSF10* (TRAIL), 5′‐GAGTATGAACAGCCCCT‐3′ and 5′‐GTTGCTTCTTCCTCTGGT‐3′.^15^, ^16^, ^17^ The endogenous genes HSP90AB1, 5′‐CCTCACTAATGACTGGGAAGAC‐3′ and *HPRT1*, 5′‐GCTTTCCTTGGTCA GGCAGTA‐3′ were obtained from a custom assay (Fluidigm, Plate ID GE_11n_1034).

The qPCR conditions and primer concentrations were validated for optimal specificity and amplification efficiency. The qPCR reaction was performed in a 10 μL volume, using SYBR Green PCR Master Mix (Cat. No. 01211573, ThermoFisher Scientific, Waltham, MA, USA), specific primers, cDNA template and nuclease‐free water. The thermal cycling conditions were an initial denaturation at 95°C for 10 min, followed by 40 cycles of 95°C for 15 s and 60°C for 1 min, with a final melting curve to verify the specificity of the amplified products. Amplification and dissociation curves were analysed to determine Ct values and compare the relative expression of the genes of interest using the ΔΔCt method, normalizing with the endogenous genes *HSP90AB1* and *HPRT1*, and relative to the control group (HD).

### 2.8 Statistical analysis

The Shapiro–Wilk normality test was used to evaluate the data distribution, indicating that our data did not follow a normal distribution. Comparisons between two groups were performed using the Mann–Whitney *U* test, while multiple comparisons were conducted using the Kruskal–Wallis test, followed by Dunn's post hoc test for correction. To analyse differences between groups of interest, statistical significance for dichotomous variables was assessed using the chi‐squared (*χ* ^2^) test. Data are presented as mean ± standard deviation (SD), and a *p*‐value of <0.05 was considered statistically significant. All statistical analyses were conducted using GraphPad Prism 10 (GraphPad Software, Inc., San Diego, CA, USA).

## 3 RESULTS

### 3.1 Description of study groups

HD and COVID‐19 patients exhibited differences in some demographic characteristics. Table 1 summarizes the demographic and laboratory data for the study groups. The severe group was significantly older than the mild and moderate groups (*p* < 0.001), and overweight and obesity were more prevalent in the severe group compared to the mild group (*p* = 0.0015). Regarding comorbidities such as diabetes and hypertension, significant differences were observed between the mild and severe groups, with the severe group serving as a reference for unfavourable outcomes.

**TABLE 1:** Demographic data of study groups.

Demographic characteristics					
	Healthy donor *n* = 30	Mild *n* = 32	Moderate *n* = 28	Severe *n* = 37	*p* value
Age (years)^d^	48 (8.3)	43 (15.7)	59 (16.8)	61 (15.7)	<0.001^a^, ^c^ <0.001^a^, ^b^
Sex^e^					
Male	15 (50)	22 (69)	17 (61)	21 (57)	ns
Female	15 (50)	10 (31)	11 (39)	16 (43)	ns
Comorbidities^e^					
Hypertension	–	8 (25)	8 (29)	19 (51)	0.0002^a^, ^c^ 0.002^b^, ^c^
Diabetes mellitus type 2	–	5 (16)	12 (43)	17 (46)	<0.001^a^, ^b^ <0.001^a^, ^c^
Overweight and obesity	–	18 (56)	20 (71)	29 (78)	0.039^a^, ^b^ 0.0015^a^, ^c^
Smoking^e^	–	12 (38)	1 (4)	13 (35)	ns

Abbreviation: ns, not significant.

^a^ Mild.

^b^ Moderate.

^c^ Severe.

^d^ Mean (SD).

^e^ *n* (%).

We observed significant differences between the mild and severe groups regarding immune cells and serologic markers associated with severity and hyperinflammation, such as lymphocytes, neutrophils, fibrinogen and C‐reactive protein (Table 2). Our computed tomography (CT) scan findings indicated that severely affected patients display pronounced lung alterations, including ground‐glass opacities, consolidation and crazy‐paving patterns. These radiologic features visually illustrate the lung damage associated with increased disease severity. Severely affected patients have more extended hospital stays, but these are not statistically different from mildly affected patients (Table S1). However, significant differences were observed between the groups in other aspects, such as mortality and the need for invasive procedures.

**TABLE 2:** Clinical and laboratory features.

Clinical and laboratory features				
	Mild	Moderate	Severe	*p* value
Blood‐routine parameters^d^				
Leucocytes (RV 4.9–10.9 × 103/mm^3^)	8 (3.3–13.9)	9 (3.4–26.5)	10 (2.1–25.8)	ns
Lymphocytes (RV 21%–48%)	(6–56.7)	(6.8–38.4)	(3.8–28.5)	0.004^a^, ^c^
Neutrophils (RV 39%–68%)	67 (9.14–91.4)	76 (56.4–96.1)	83 (67.1–93.2)	0.007^a^, ^c^
Platelet (RV 175–378 × 103/mm^3^)	258 (82–587)	267 (94–545)	204 (52–330)	0.038^a^, ^c^ 0.017^b^, ^c^
Lactate dehydrogenase (RV 140–271 U/L)	420 (124–1265)	501 (156–2002)	676 (234–1835)	0.011^a^, ^c^
Creatinine (RV 0.6–1.2 mg/dL)	1 (0.4–2.34)	5 (0.4–81.3)	1 (0.42–2.5)	ns
D‐dimer (RV 0–600 ng/mL)	691 (97–5000)	1605 (94–5604)	1361 (98–6520)	ns
Fibrinogen (RV 238–498 mg/dL)	491 (194–916)	639 (156–972)	568 (161–1023)	0.031^a^, ^c^
C‐Reactive protein (RV 1–10 mg/dL)	64 (0–270)	124 (15.3–358.7)	124 (10.6–542.5)	0.024^a^, ^c^ 0.0407^a^, ^b^
Glucose (RV 74–106 mg/dL)	138 (89–367)	172 (51–401)	152 (59–456)	ns
Ferritin (RV 12–300 ng/mL)	421 (40.5–1520)	507 (119–1610)	924 (210–7950)	ns
Chest computed tomography (CT)^e^				
Ground glass opacity	19 (60)	5 (18)	8 (21)	<0.001^a^, ^b^ <0.001^a^, ^c^
Consolidation	1 (3)	11 (39)	14 (38)	<0.001^a^, ^b^ <0.001^a^, ^c^
Crazy‐paving	1 (3)	4 (14)	4 (11)	0.0093^a^, ^b^ 0.048^a^, ^c^
No CT scan	11 (34)	8 (29)	11 (30)	ns

Abbreviations: ns, not significant; RV, reference value provided by the clinical laboratory.

^a^ Mild.

^b^ Moderate.

^c^ Severe.

^d^ Mean (min, max).

^e^ *n* (%).

Considering the large diversity of symptoms and treatments, a donut‐type parts‐of‐whole graph was created to visualize them. No significant disparities were observed between groups; results indicate similar proportions of symptoms (Figure S1, upper panel). Regarding pharmacological treatments, the distribution of treatments is quite similar among groups, except that baricitinib and tocilizumab were not used in mildly affected patients; the first was used in some moderately affected and severely affected patients and the second only in severe cases (Figure S1, lower panel).

### 3.2 TRAIL is decreased in the serum of COVID‐19 patients, particularly those with severe conditions

Reports indicate that severe COVID‐19 cases display high systemic levels of molecules associated with cell death via the TNFR1 pathway.^13^, ^14^ Using a LEGENDplex™ assay, we measured systemic protein levels of TNFSF members to identify the dynamics of these molecules and their potential relation to specific clinical conditions.

**FIGURE 1:**
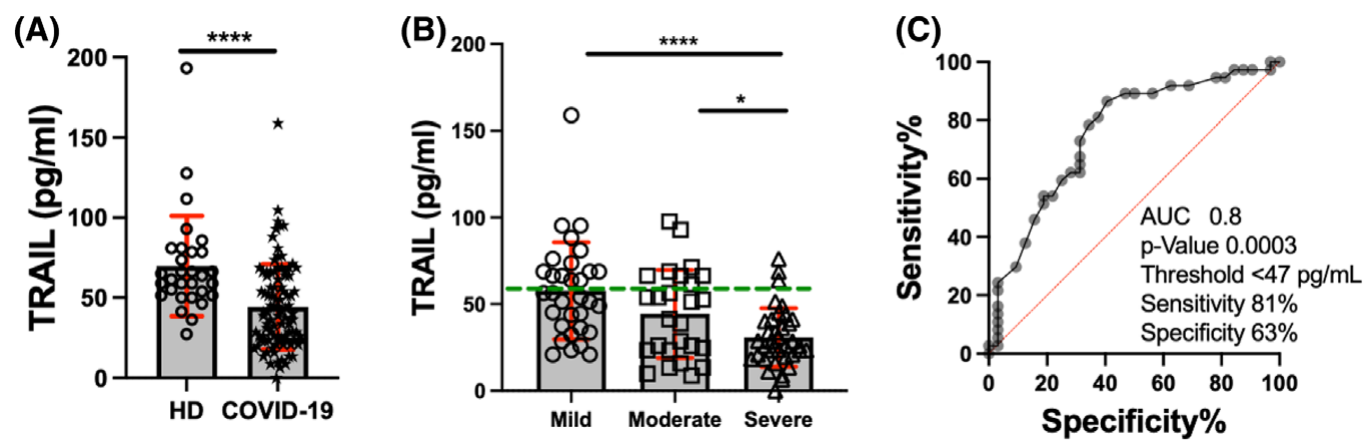
Systemic TRAIL levels are decreased in COVID‐19 patients. LEGENDplex™‐evaluated serum TRAIL levels; a comparison between healthy donors (HD, *n* = 30) and COVID‐19 (*n* = 98) was performed (A). TRAIL levels were compared among mild (*n* = 32), moderate (*n* = 29) and severe (*n* = 37) COVID‐19 groups of patients (B); the dotted green line indicates the mean HD value. Data are presented as mean +/− SD (red line); each symbol represents an individual subject. The receiver operating characteristics (ROC) curve shows the area under the curve (AUC), which is identified by comparing mild (dotted red line) and severe groups (grey dot). Sensitivity, specificity, and cut‐off value (pg/mL) were obtained (C). Statistical analysis was performed with the Mann–Whitney *U* test to compare two groups (to A) or the Kruskal–Wallis test and corrected using Dunn's test to compare among groups (to B). ****p < 0.0001, *p < 0.05.

Our research identified a significant decrease in TRAIL protein in COVID‐19 patients who showed lower soluble TRAIL levels than HD (45 pg/mL vs. 70 pg/mL, *p* < 0.0001) (Figure 1A). The severe group, in particular, had lower TRAIL levels than both the mild and moderate groups (severe: 31 pg/mL, mild: 58 pg/mL, *p* < 0.0001; moderate: 44 pg/mL, *p* < 0.05) (Figure 1B).

These findings show that systemic TRAIL levels are affected during COVID‐19 and suggest that TRAIL protein levels are directly related to the severity of the condition. Our evaluation of TRAIL levels as a potential differentiator between mild (dotted red line) and severe (grey dots) COVID‐19 cases further supports this notion. TRAIL exhibited an AUC of 0.8 (95% CI = 0.68–1.0) with a cut‐off value lower than 47 pg/mL, yielding a sensitivity and specificity of 81% and 63%, respectively (Figure 1C). These results were confirmed by an ELISA assay, where the same TRAIL profile was detected, characterized by low TRAIL levels in patients severely affected by COVID‐19 (Figure S2).

These data indicate that low TRAIL protein levels could be a valuable clinical tool for identifying severe conditions.

### 3.3 High systemic levels of OPG and BAFF are associated with the severity of COVID‐19

**FIGURE 2:**
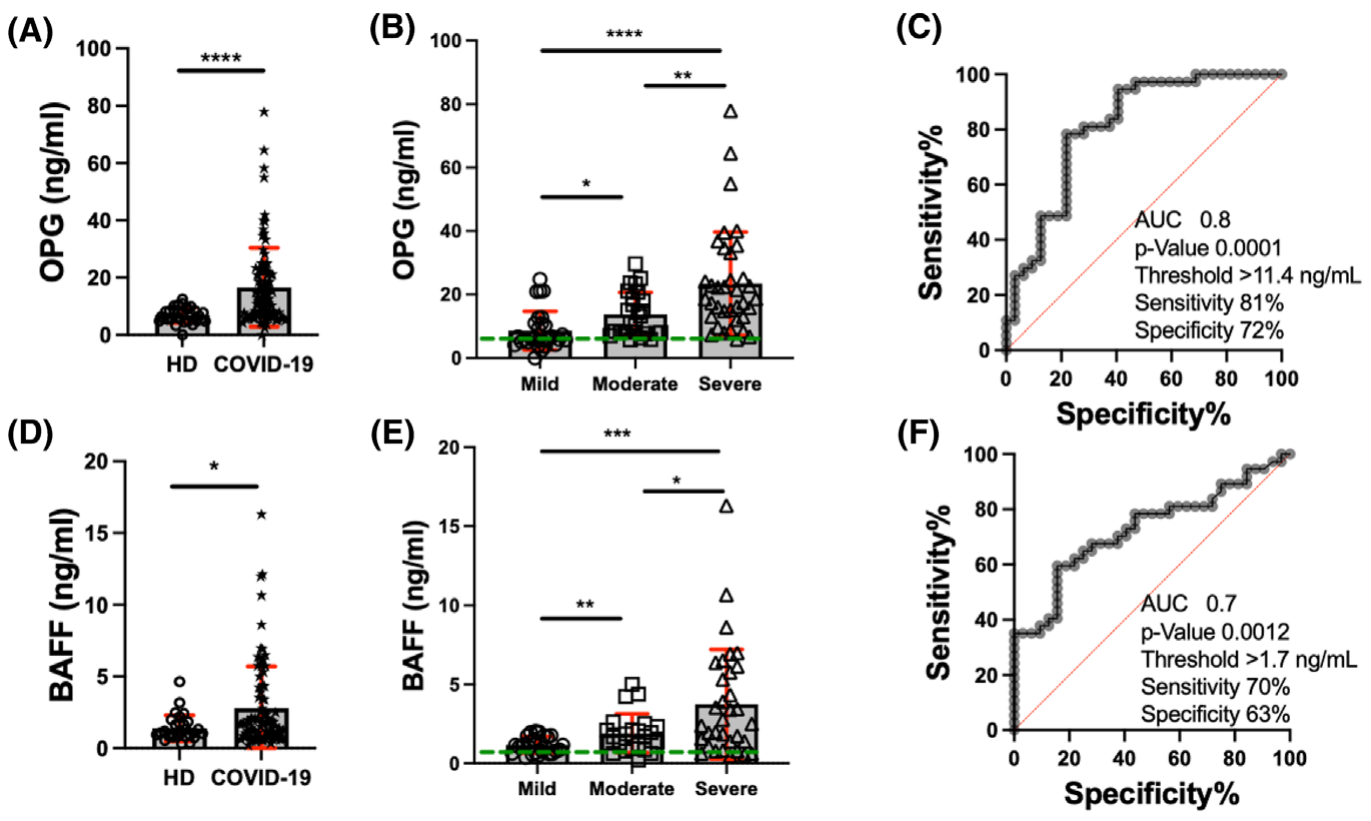
Systemic levels of OPG and BAFF are increased in severe COVID‐19 cases. OPG and BAFF levels were evaluated in sera of healthy donors (HD) and COVID‐19 patients by LEGENDplex™. OPG levels were compared between HD (*n* = 30) and COVID‐19 (*n* = 98) (A) and among mildly (*n* = 32), moderately (*n* = 29) and severely (*n* = 37) COVID‐19 affected patients (B). Similarly, BAFF levels between HD (*n* = 30) and COVID‐19 (*n* = 98) cases were compared (D). BAFF levels were also compared among mild (*n* = 32), moderate (*n* = 29) and severe (*n* = 37) COVID‐19 cases (E), where the dotted green line indicates the mean HD value. Data are presented as mean +/− SD (red); each symbol represents an individual subject. The receiver operating characteristics (ROC) curves show the area under the curve (AUC) identified by comparing mild (dotted red line) and severe cases (grey dot); then, the sensitivity, specificity and cut‐off value (pg/mL) were obtained for OPG (C) and BAFF (F). Statistical analysis was performed with the Mann–Whitney *U* test to compare two groups (A and D) or the Kruskal–Wallis test and corrected using Dunn's test to compare groups (B and E). ****p < 0.0001, ***p < 0.001, **p < 0.01 and *p < 0.05.

LEGENDplex™ results indicate that systemic OPG levels are increased in COVID‐19 patients compared to HD (18.3 ng/mL vs. 6.8 ng/mL, *p* < 0.0001) (Figure 2A); among them, severe cases display higher OPG levels (severe 23.4 ng/mL) than mild or moderate cases (9.2 ng/mL, *p* < 0.0001; 13.7 ng/mL, *p* < 0.01) (Figure 2B), and moderate cases have higher levels than mild cases (*p* < 0.05). Moreover, mild COVID‐19 cases (dotted red‐line) were compared to severe (grey dots), and we observed that OPG has an AUC of 0.8 (95% CI = 0.68–1.0) with a cut‐off of 11.4 ng/mL, and a sensitivity and specificity of 81% and 72%, respectively (Figure 2C).

A similar profile is observed for systemic BAFF levels; COVID‐19 patients have higher BAFF levels compared to HD (2.7 ng/mL vs. 1.3 ng/mL, *p* < 0.05) (Figure 2D), and also, severe cases display higher levels (severe 3.7 ng/mL) than mild or moderate cases (1.1 ng/mL, *p* < 0.001; 1.9 ng/mL, *p* < 0.05) (Figure 2E), and moderate cases have higher levels than mild cases (*p* < 0.01). BAFF has an AUC of 0.7 (95% CI = 0.68–1.0) with a cut‐off of 1.7 ng/mL and a sensitivity and specificity of 70% and 63%, respectively (Figure 2F).

These significant findings underscore the crucial role of high levels of OPG, BAFF, and low levels of TRAIL in the severity of COVID‐19. Notably, OPG demonstrates superior sensitivity and specificity when severely affected compared to mildly affected patients, further emphasizing its potential as a biomarker for disease severity.

### 3.4 Systemic APRIL, LIGHT, CD30L and CD40L levels increase in COVID‐19 patients but do not differ based on severity status

**FIGURE 3:**
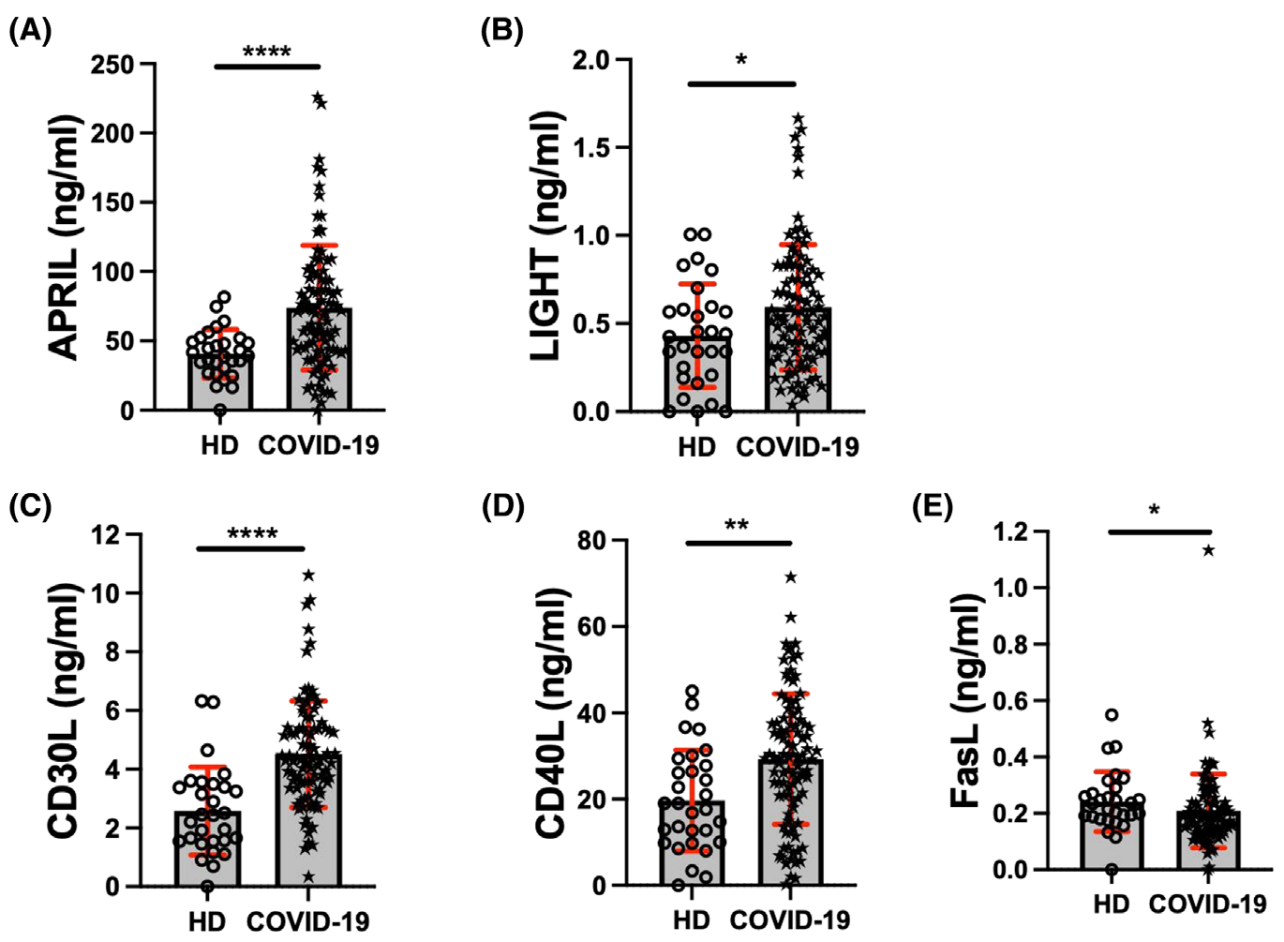
APRIL, LIGHT, CD30L and CD40L are elevated in sera from COVID‐19 patients. APRIL, LIGHT, CD30L, CD40L and FasL (A–E, respectively) levels were evaluated in sera of healthy donors (HD) (*n* = 30) and COVID‐19 patients (*n* = 98) by LEGENDplexTM. Data are presented as mean +/− SD (red); each symbol represents an individual subject. Statistical analysis was performed using the Mann–Whitney *U* test to compare the two groups. ****p < 0.0001, **p < 0.01 and *p < 0.05.

Patients with COVID‐19 show significantly higher protein levels of APRIL (COVID‐19: 77.4 ng/mL vs. HD: 40.7 ng/mL, *p* < 0.0001); LIGHT (COVID‐19: 0.60 pg/mL vs. HD: 0.43 ng/mL, *p* < 0.05); CD30L (COVID‐19: 4.5 ng/mL vs. HD: 2.5 ng/mL, *p* < 0.0001); and CD40L (COVID‐19: 29.3 ng/mL vs. HD: 19.6 ng/mL, *p* < 0.01) (Figure 3A–D, respectively). On the contrary, systemic FasL levels are lower in COVID‐19 patients (COVID‐19: 0.20 ng/mL vs. HD: 0.24 ng/mL, *p* < 0.05) (Figure 3E).

Although all these molecules showed marked differences between COVID‐19 patients and HD when patients are divided into clinical status, levels of these molecules are not different (Figure S3).

### 3.5 TNF‐α, TNF‐β, RANKL and TWEAK serum levels are not modified in patients with COVID‐19

**FIGURE 4:**
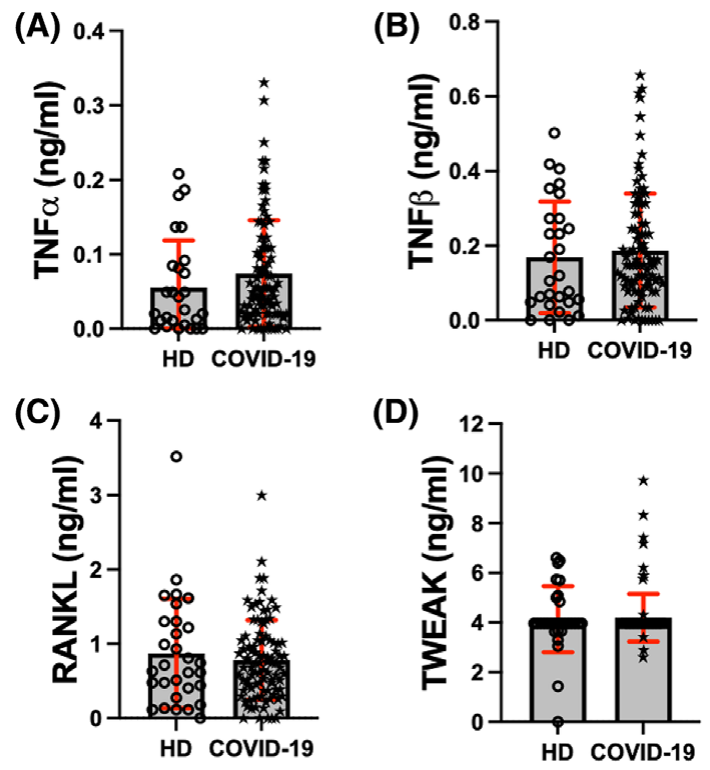
TNF‐α, TNF‐β, RANKL and TWEAK serum levels show no differences between COVID‐19 patients and HD. LEGENDplex™ evaluated TNF‐α, TNF‐β, RANKL and TWEAK (A–D, respectively) levels in sera obtained from healthy donors (HD) (*n* = 30) and COVID‐19 patients (*n* = 98). Data are presented as mean +/− SD (red); each symbol represents an individual subject.

As illustrated in Figure 4A–D, serum levels of other TNF‐family members, such as TNF‐α, TNF‐β RANKL and TWEAK, do not display differences between HD and COVID‐19 patients. In line with this, when COVID‐19 patients are ranked by severity, TNF‐α, TNF‐β, RANKL and TWEAK are similar across groups (Figure S4).

### 3.6 Transcriptional levels of TRAIL, BAFF and OPG increase across mild to severe COVID‐19 cases

Our results indicate that TRAIL, BAFF and OPG systemic levels could be used as severity biomarkers in COVID‐19. To confirm that the TRAIL, BAFF and OPG protein expression pattern follows their transcript expression, the transcript levels for these three molecules were evaluated in mRNA obtained from peripheral blood mononuclear cells in patients ranging from severely to mildly affected by COVID‐19.

**FIGURE 5:**
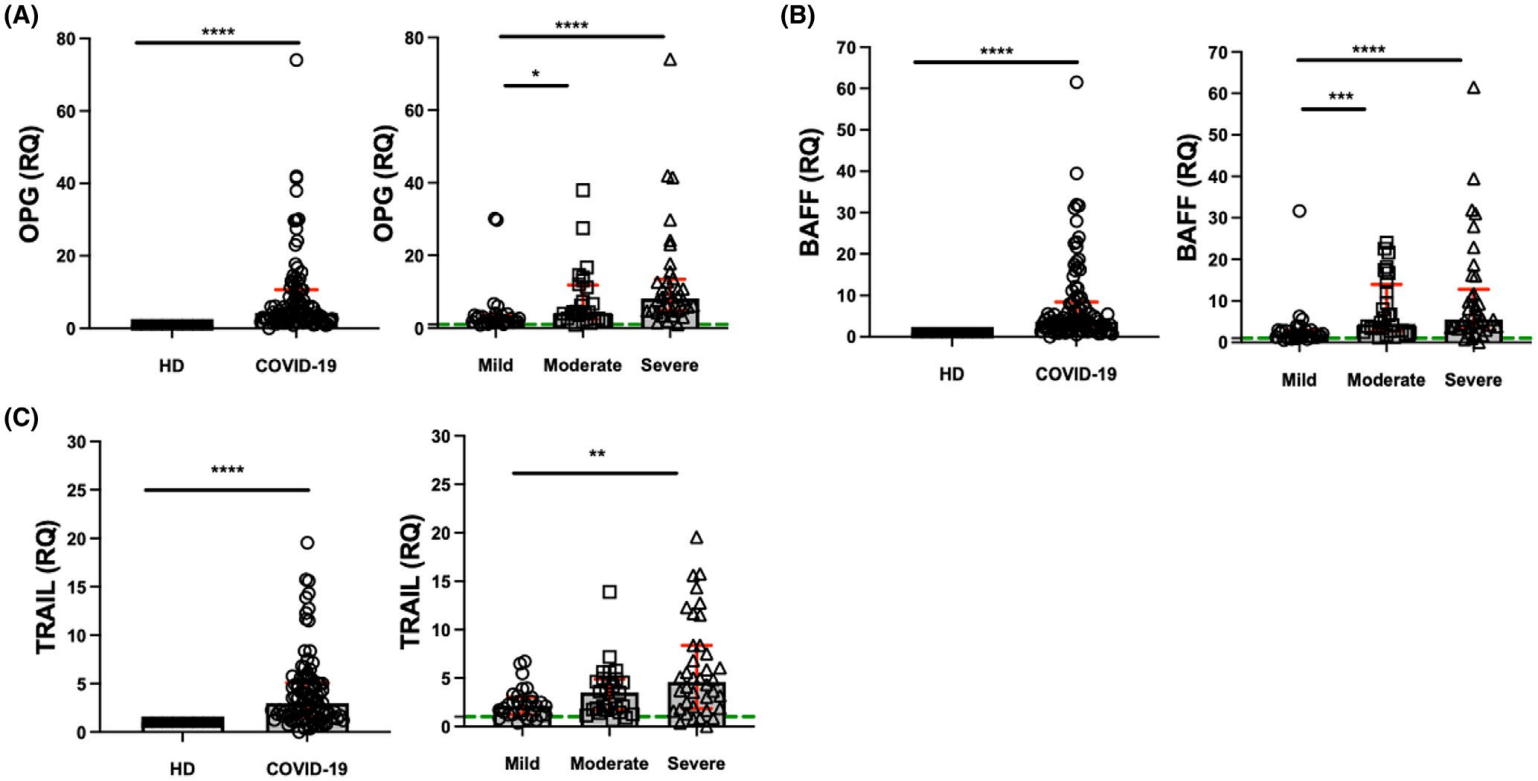
Transcriptional levels of TRAIL, OPG, and BAFF in COVID‐19 patients. OPG, BAFF and TRAIL (A–C, respectively) levels were evaluated using qPCR in healthy donors (HD) and COVID‐19 patients. OPG levels significantly differ between HD and COVID‐19, mildly affected and moderately affected, and mildly affected and severely affected patients. BAFF levels significantly differ between HD and COVID‐19, mildly affected and moderately affected, and mildly affected and severely affected patients. TRAIL levels significantly differ between HD and COVID‐19 and mild and severely affected patients. Data are presented as mean +/− SD (red); each symbol represents an individual subject, and HD levels were normalized to 1 as a reference. Statistical analysis was performed with the Mann–Whitney *U* test to compare two groups (A–C, left) or the Kruskal–Wallis test and corrected using Dunn's test to compare between groups (A–C, right). ****p < 0.0001, ***p < 0.001, **p < 0.01 and *p < 0.05.

Confirming our results obtained at the protein level, OPG mRNA levels (*p* < 0.0001) and BAFF mRNA levels (*p* < 0.0001) increased in COVID‐19 patients when compared to HD (Figure 5A,B, left, respectively). A similar increasing trend was observed for both OPG and BAFF mRNA levels when comparing severely affected (*p* < 0.0001) with moderately affected and moderately affected (*p* < 0.001) with mildly affected patients (Figure 5A,B, right, respectively).

In contrast, unlike what was observed with the soluble form, TRAIL mRNA expression increases in COVID‐19 patients compared to HD (*p* < 0.0001). Moreover, while severely affected patients show significantly higher levels than mildly affected patients (*p* < 0.01), the change was not significant when comparing severely affected to moderately affected patients (Figure 5C).

## 4 DISCUSSION

The results of this study unveil interesting changes in the serum concentration of several TNF/TNFR superfamily members in patients with COVID‐19. The concomitant increase of OPG (osteoprotegerin) and BAFF (B‐cell activating factor), particularly in severe cases, underscores the distinctive immunological dysfunction observed in COVID‐19. These molecules, which regulate B lymphocytes and the adaptive immune response, may play a key role in the development of autoimmunity and exacerbated inflammation. The notable increase in BAFF, known for inducing Th1 lymphocyte responses and triggering inflammation, suggests that it contributes to the uncontrolled immune response in some COVID‐19 patients.^11^, ^18^

Similarly, OPG and BAFF transcriptional levels are significantly elevated in COVID‐19 patients compared to HD, with the severely and moderately affected groups showing higher levels than mildly affected cases. This consistent increase across both protein and mRNA levels demonstrates their potential as biomarkers for disease severity.

To support our hypothesis, an additional existing COVID‐19‐related gene expression dataset (https://covidgenes.weill.cornell.edu/), which is available through an online platform and contains a large cohort of vaccination‐naïve individuals (*n* = 735), was analysed. We found that mRNA levels for TRAIL and BAFF are elevated in the nasopharyngeal swab transcriptome obtained from 216 COVID‐19 positive compared with 519 COVID‐19 negative,^19^, ^20^ and more interestingly, this increase was associated with the viral load (Figure S5). Regarding OPG, we did not see a similar trend in these analyses, likely due to the low resolution of high‐throughput RNA sequencing and the low levels of OPG gene expression. In consonance, a previous report indicates prominent BAFF and TRAIL gene expression in lung areas of severe diffuse alveolar damage.^21^

Our findings were further supported by RNA sequencing data from the PREDICT‐19 consortium (GSE217948, PMID: 36741372). Analysis of this dataset revealed a significant increase in TRAIL and BAFF transcript levels in COVID‐19‐positive patients compared to HDs (control), while OPG levels remained unchanged (Figure S6A). Similar results were obtained when comparing RNA sequencing data from another study (GSE172114, PMID: 34698500) between critical and non‐critical COVID‐19 patients (Figure S6B). Notably, OPG levels were nearly undetectable in most samples from both datasets; the measurement of OPG by qPCR, as we did in our study, was more accurate and sensitive for detecting the changes in gene expression due to SARS‐CoV‐2 infection.

The discovery of significantly reduced serum levels of TRAIL despite higher levels of transcription in patients with COVID‐19, particularly in severe cases, is an unexpected finding that raises essential questions about the role of this molecule in disease pathogenesis. TRAIL, known for regulating apoptosis and the immune response, is crucial in viral evasion and inflammation associated with disease severity. The decline in TRAIL could promote the persistence of infected cells and intensify the inflammatory response, underscoring its importance in the complex network of immunological interactions in COVID‐19.^22^

In cancer cells, TRAIL, produced by immune cells, has been shown to induce apoptosis of tumour cells through its interaction with death receptors TRAIL‐R1 and TRAIL‐R2, but TRAIL‐R4 can act as a decoy receptor, inhibiting TRAIL‐induced apoptosis and contributing to tumour cell survival.^23^ Also, in cancer cells of epithelial origin, it has been shown that TRAIL RNA levels increase upon IFN stimulation; however, soluble TRAIL protein levels do not change. Instead, TRAIL protein accumulates intracellularly in these cells, suggesting a mechanism cancer cells use to avoid autocrine or paracrine‐induced apoptosis.^24^ This mechanism could similarly be employed in cases of severe COVID‐19, thus reducing cell death by apoptosis and sustaining the survival of infected cells.

Overall, our findings and existing knowledge about TRAIL in infection and cancer suggest that TRAIL's role in immune regulation and tumour surveillance is complex, multifaceted, and extends beyond cell death. Therefore, TRAIL in severely affected COVID‐19 patients may work as a molecule of immune evasion and resistance to apoptosis.

The elevation of APRIL, LIGHT, CD30L and CD40L and the decrease of FasL in COVID‐19 patients indicate the activation of the immune system and inflammation associated with the disease. These molecules, implicated in regulating the immune response and inflammation, could contribute to the observed pathology. Excessive immune activation, as suggested by the elevation of these molecules, highlights the immune response's complexity to SARS‐CoV‐2 infection.^25^, ^26^

On the other hand, serum levels of TNF‐α, TNF‐β, RANKL and TWEAK showed no significant differences between COVID‐19 patient groups and HDs, suggesting that these particular cytokines may not be as critical in the differential immune response or progression of COVID‐19 as previously thought.^27^, ^28^

This study is not exempt from limitations; for instance, we do not have a long‐term follow‐up of patients, and our cohort does not have the viral load data, so it is impossible to confirm in our patients the correlation between high OPG, BAFF and TRAIL levels with viral load, a result provided by analysis of a public database. However, despite these limitations, our study highlights the potential biomarkers for severity in COVID‐19 patients and the necessity for additional research to validate and expand these findings.

## 5 CONCLUSION

This study provides detailed insight into the immune response in patients with COVID‐19, emphasizing the importance of several key biomarkers in the pathogenesis of the disease. Our study suggests that the elevated OPG and BAFF levels at both protein and mRNA levels underline their potential as biomarkers for disease severity. Moreover, the decreased soluble TRAIL and increased transcriptional levels suggest a complex regulatory mechanism.

## AUTHOR CONTRIBUTIONS

**Andy Ruiz:** Conceptualization (equal); formal analysis (equal); investigation (equal); methodology (equal); writing – original draft (equal). **Carlos Peña Bates:** Conceptualization (equal); formal analysis (equal); investigation (equal); methodology (equal); writing – original draft (equal). **Lucero A. Ramon‐Luing:** Methodology (equal); writing – review and editing (equal). **Daniel Baca‐Nuñez:** Methodology (equal); writing – review and editing (equal). **Marco Antonio Vargas:** Data curation (equal); writing – review and editing (equal). **Karen Medina‐Quero:** Data curation (equal); writing – review and editing (equal). **Neptali Gutierrez:** Data curation (equal); writing – review and editing (equal). **Joel A. Vázquez‐Pérez:** Resources (equal); writing – review and editing (equal). **Ramcés Falfán‐Valencia:** Formal analysis (equal); writing – review and editing (equal). **Gloria Pérez‐Rubio:** Formal analysis (equal); writing – review and editing (equal). **Carolina Di Benedetto:** Formal analysis (equal); writing – review and editing (equal). **Ivette Buendia‐Roldan:** Data curation (equal); writing – review and editing (equal). **Moisés Selman:** Investigation (equal); writing – review and editing (equal). **Paola Betancur:** Conceptualization (equal); funding acquisition (equal); supervision (equal); writing – review and editing (equal). **Leslie Chavez‐Galan:** Conceptualization (equal); formal analysis (equal); funding acquisition (equal); investigation (equal); project administration (equal); supervision (equal); validation (equal); writing – original draft (equal); writing – review and editing (equal).

## FUNDING INFORMATION

LCG received financial support from research funds of the Instituto Nacional de Enfermedades Respiratorias Ismael Cosío Villegas (INER) and of Dirección General de Políticas en Investigación en Salud, Ministry of Health of Mexico, grant number FPIS‐2024‐4810. PB received financial support from the COVID Catalyst Award Henry Wheeler Center for Emerging Neglected Diseases (CEND).

## CONFLICT OF INTEREST STATEMENT

The authors declare no conflicts of interest.

## Supporting information


Figure S1.
Figure S2.
Figure S3.
Figure S4.
Table S1.

## Data Availability

The datasets generated for this study are available from the corresponding author (Leslie Chavez‐Galan) upon reasonable request.
