# Supplementary material for: OPG and BAFF as predictive biomarkers of the severity of SARS‐CoV‐2 infection
Source: J Cell Mol Med. 2025 Jan 31;29(3):e70189. doi: 10.1111/jcmm.70189 (PMC11783147; doi:10.1111/jcmm.70189)
Supplement: Supplementary file 1 — Figure S1. Figure S2. Figure S3. Figure S4. Table S1. [file JCMM-29-e70189-s001.docx]

**SUPPLEMENTARY TABLES AND FIGURES**

**OPG and BAFF as predictive biomarkers in the progression of SARS-CoV-2 infection**

Andy Ruiz^1†^, Carlos Peña Bates^1†^, Lucero A. Ramon-Luing^1^, Daniel Baca-Nuñez ^2^, Marco Antonio Vargas^2^, Karen Medina-Quero^2^, Neptali Gutierrez^2^, Joel A. Vázquez-Pérez^1^, Ramcés Falfán-Valencia^1^, Gloria Pérez-Rubio^1^, Carolina Di Benedetto^3^, Ivette Buendia-Roldan^1^, Moisés Selman^1^, Paola Betancur^3*^, Leslie Chavez-Galan^1*^

^†^ These authors contributed equally to this work.

**Material and Methods**

**GEO dataset analysis**

For gene expression analysis was performed using RNA-sequencing information from blood samples obtained from public datasets GSE217948 (ref: PMID: 36741372) and GSE172114 (ref. PMID: 34698500). Samples from GSE217948 were grouped based on their COVID status as healthy individuals or COVID-19 positive patients. Samples from GSE172114 were grouped based on disease severity as non critical or critical COVID patients. Analysis was performed using GEO2R tool (PMID 23193258) and the Benjamini & Hochberg test for False discovery rate.

**Table S1. Disease progression**

| Disease progression | | | | |
| --- | --- | --- | --- | --- |
|  | **Mild** | **Moderate** | **Severe** | **P value** |
| Length of hospital stay (in days)* | 11(2-41) | 13(2-26) | 16(2-47) | ns |
| Admission to the intensive care unit | 1 (3) | 4 (14) | 4 (11) | 0.0093^a,b^  0.048^a,c^ |
| Mechanical ventilation | 1 (3) | 3 (11) | 7 (19) | 0.0004^a,c^ |
| Reintubation | 1 (3) | 1 (4) | 2 (5) | ns |
| Kidney damage | 1 (3) | 3 (11) | 1 (3) | 0.048^a,b^  0.048^a,c^ |
| Hospital discharge** | 28 (88) | 19 (68) | 19 (51) | 0.0010^a,b^ <0.001^a,c^  0.020^b,c^ |
| Death | 1 (3) | 5 (18) | 15 (41) | 0.0008^a,b^  <0.0001^a,c^  0.0006^b,c^ |
| No data | 3 (9) | 4(14) | 3(8) | - |

Data showed as: *mean (SD), **N (%), ^a^Mild, ^b^Moderate, ^c^Severe, ns= not significant.

**FIGURES**

**
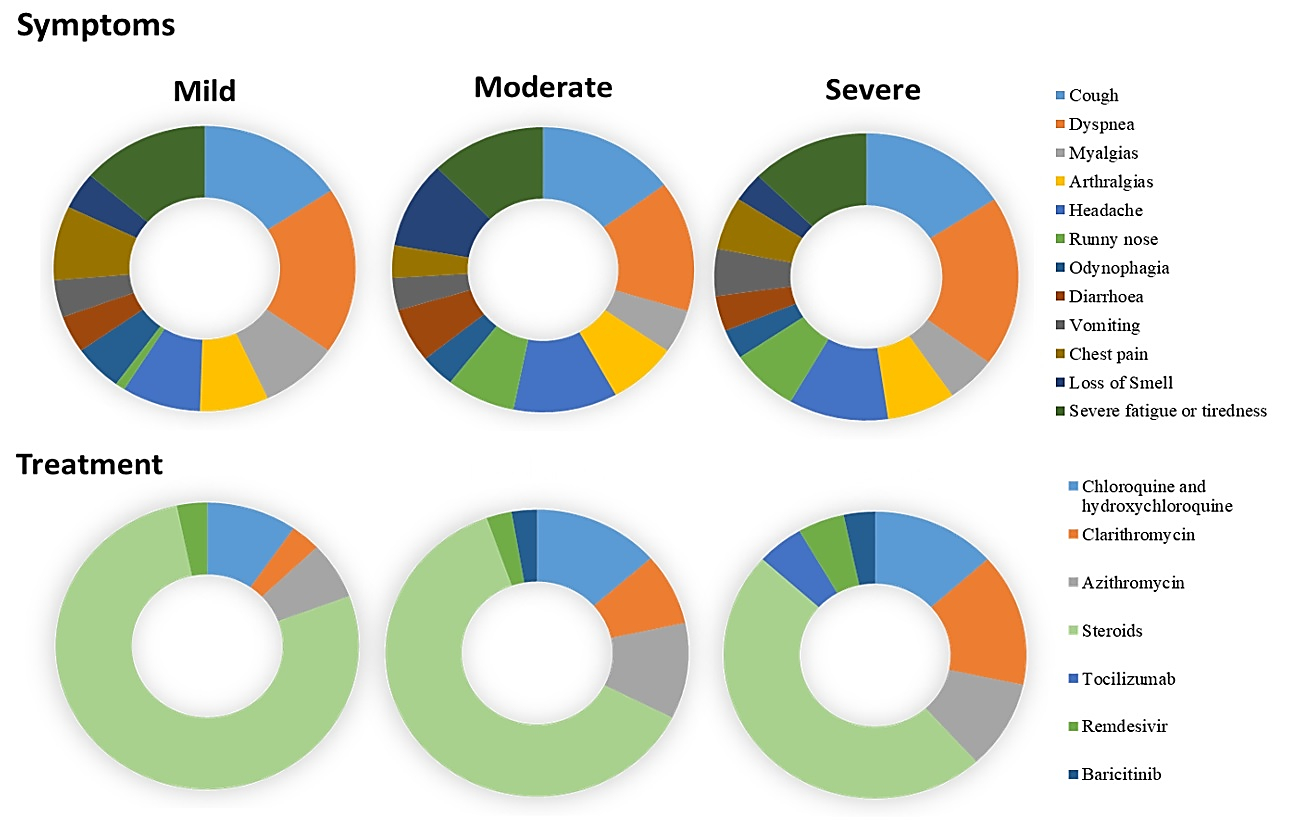
**

**Figure S1.** Prevalence of Symptoms and Pharmacological Treatments in Patients with COVID-19. The donut charts for mild (Mild), moderate (Moderate), and severe (Severe) patient groups illustrate the distribution of symptoms and treatments in each group.


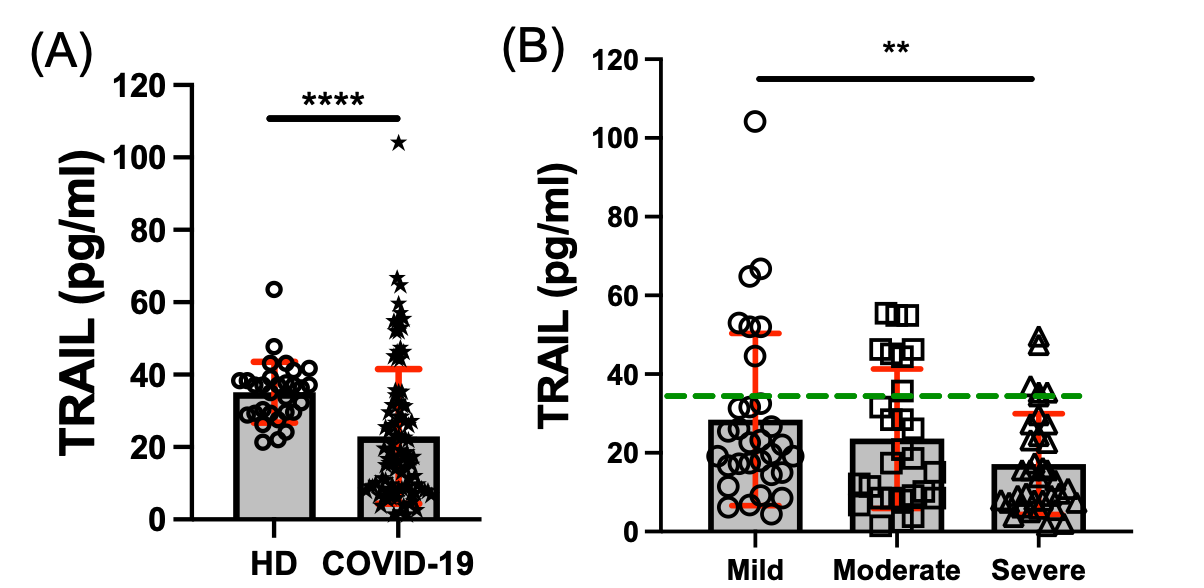


**Figure S2.** Systemic TRAIL levels decreased in COVID-19 patients, mainly in the severe status. Using an ELISA, TRAIL levels were compared between HD (n=30) and COVID-19 (n=96) (A), and among mildly (n=32), moderately (n=28), and severely (n=36) COVID-19-affected patients (B), where the dotted green line indicates the mean HD value. Data are presented as mean +/- standard deviation (red); each symbol represents an individual subject. Statistical analysis was performed with the Mann–Whitney U test to compare two groups (to A) or the Kruskal–Wallis test and corrected using Dunn’s test to compare among groups (to B). ****p< 0.0001, **p< 0.01.


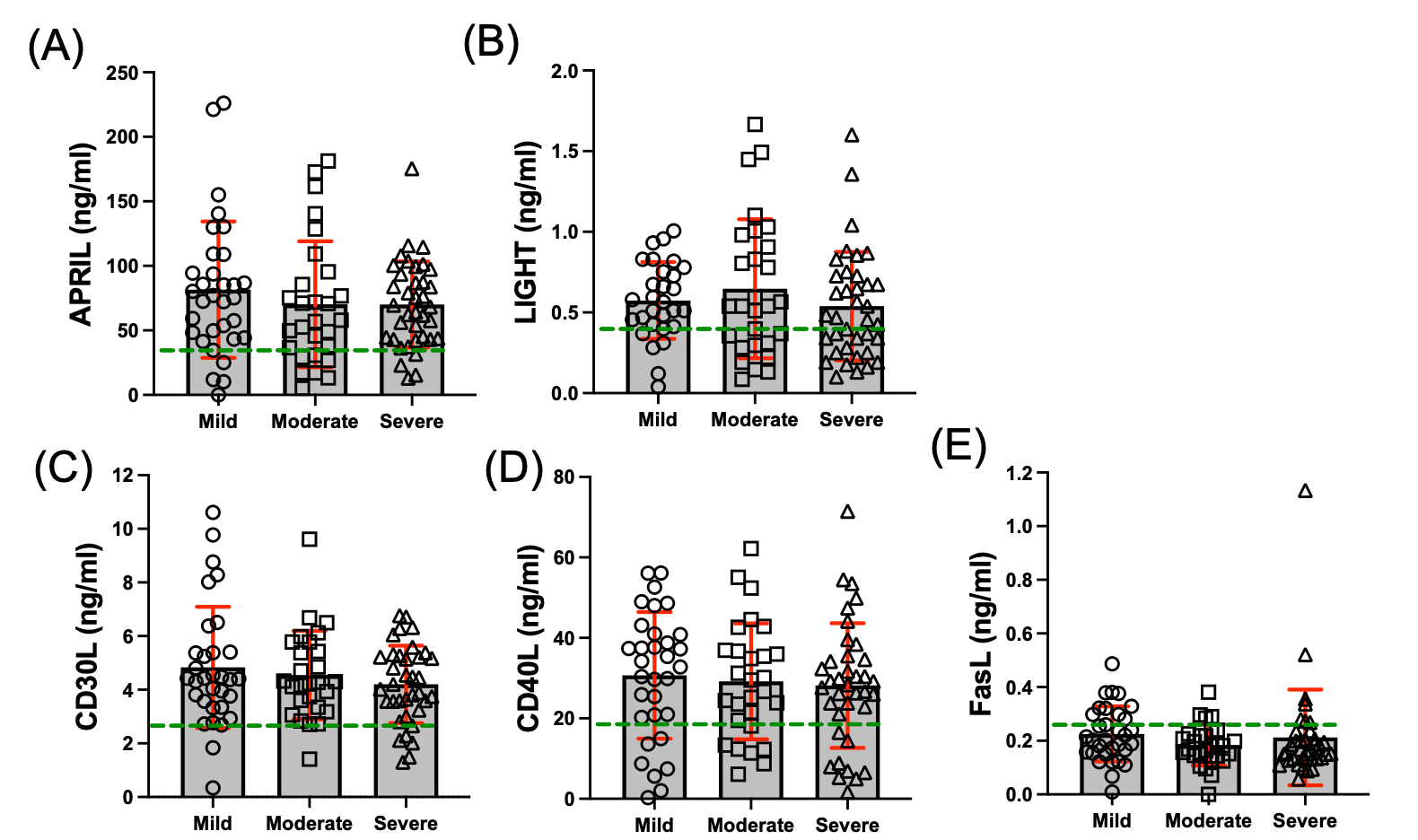


**Figure S3.** Systemic APRIL, LIGHT, CD30L, and CD40L levels in patients with COVID-19. Assessments were performed using LEGENDplexTM among mild (n=32), moderate (n=29), and severe (n=37) COVID-19 (n=98) groups. (A) APRIL, (B) LIGHT, (C) CD30L, (D) CD40L, and (E) FasL. Each symbol represents an individual. No significant differences were found among the groups using the Kruskal-Wallis test.


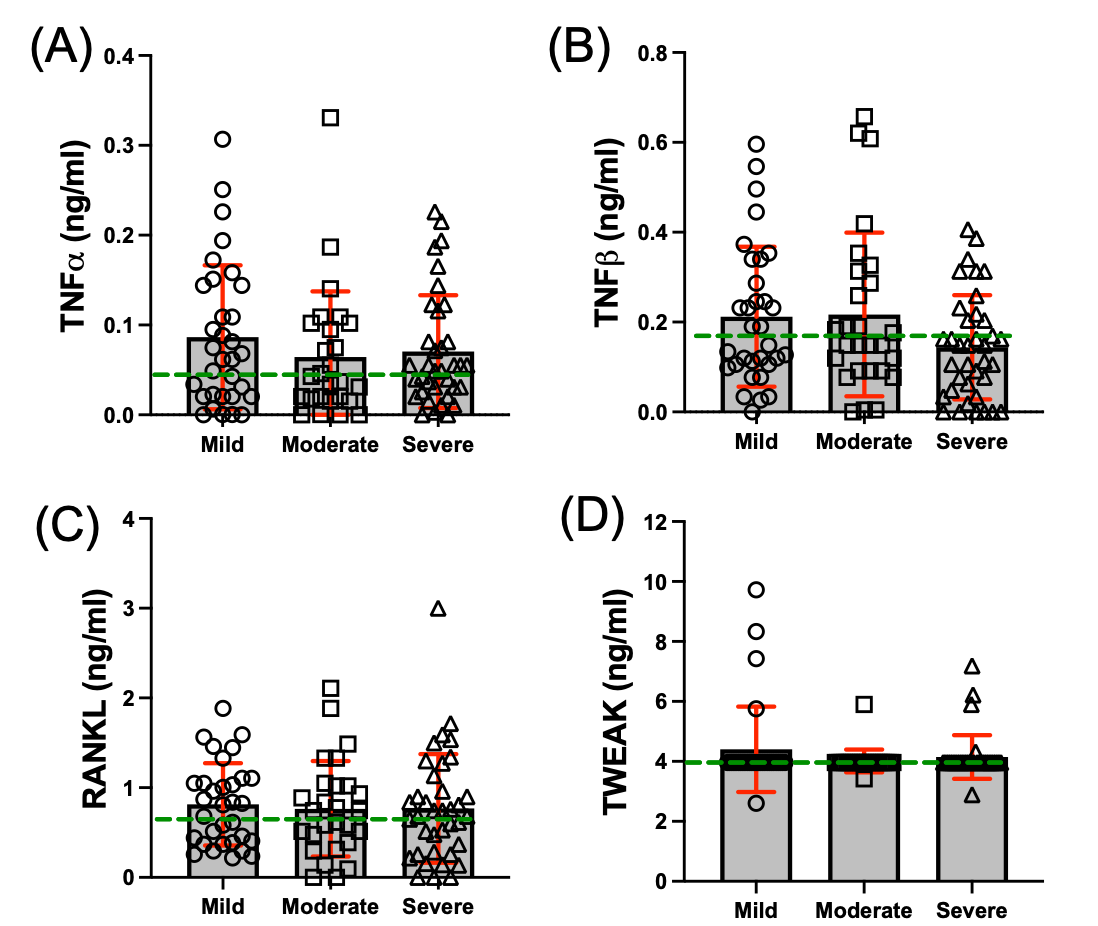


**Figure S4.** Systemic TNF-α, TNF-β, RANKL, and TWEAK levels in patients with COVID-19. Assessments were performed using LEGENDplexTM among mild (n=32), moderate (n=29), and severe (n=37) COVID-19 (n=98) groups. (A) TNF-α, (B) TNF-β, (C) RANKL, and (D) TWEAK. Each symbol represents an individual. No significant differences were found among groups using the Kruskal-Wallis test.


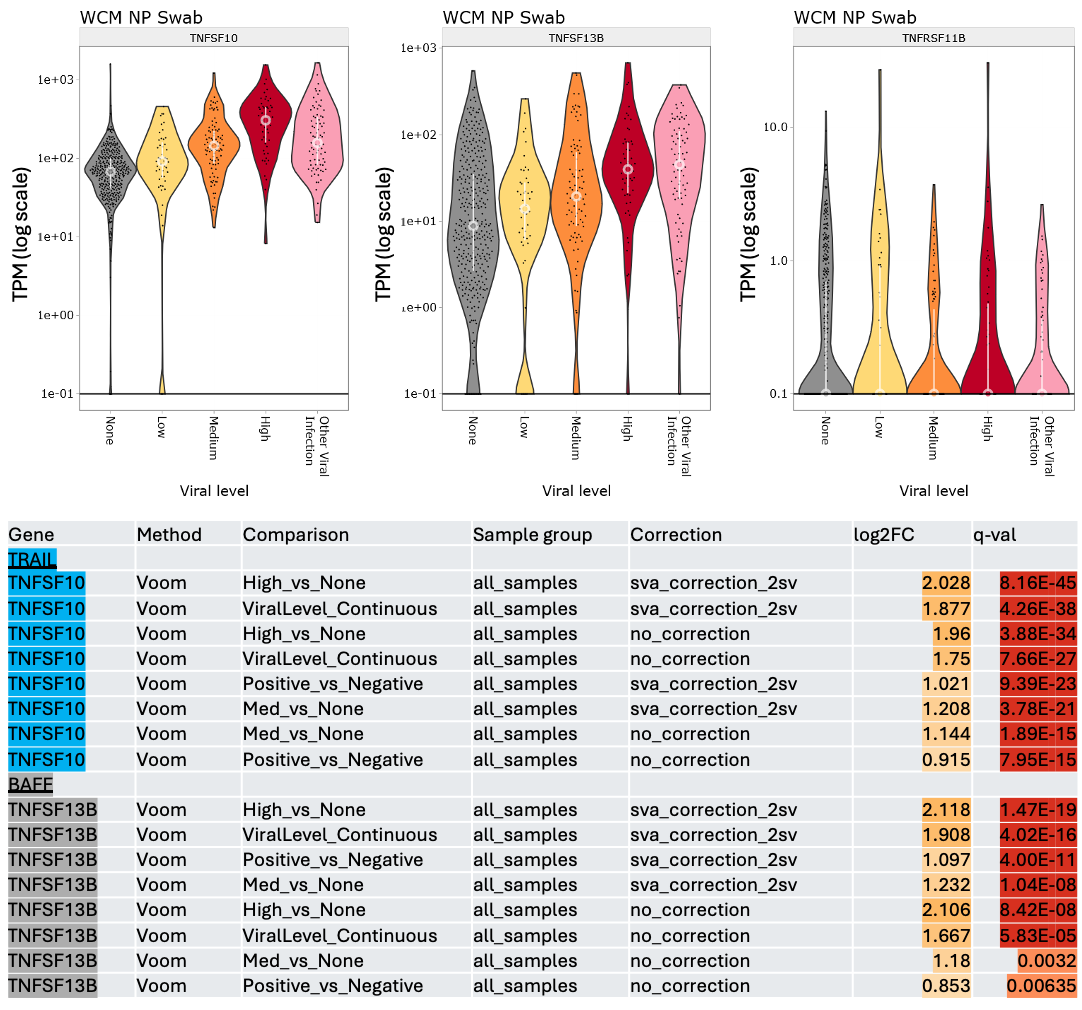


**Figure S5.** TRAIL (TNFSF10), BAFF (TNFSF13B), and OPG (TNFRSF11B) RNA expression in nasal swabs across SARS-CoV-2 viral load. In the violin graphs, the Y axis is presented as transcript per million reads mapped (TPM) in the log scale (TPM log scale), while the X axis represents viral load. Data were obtained from the website https://covidgenes.weill.cornell.edu/, and the table shows the analysis performed using the ‘differentially expressed genes (DEG) tool’. WCM NP = Weill Cornell Medicine and Nasopharyngeal.


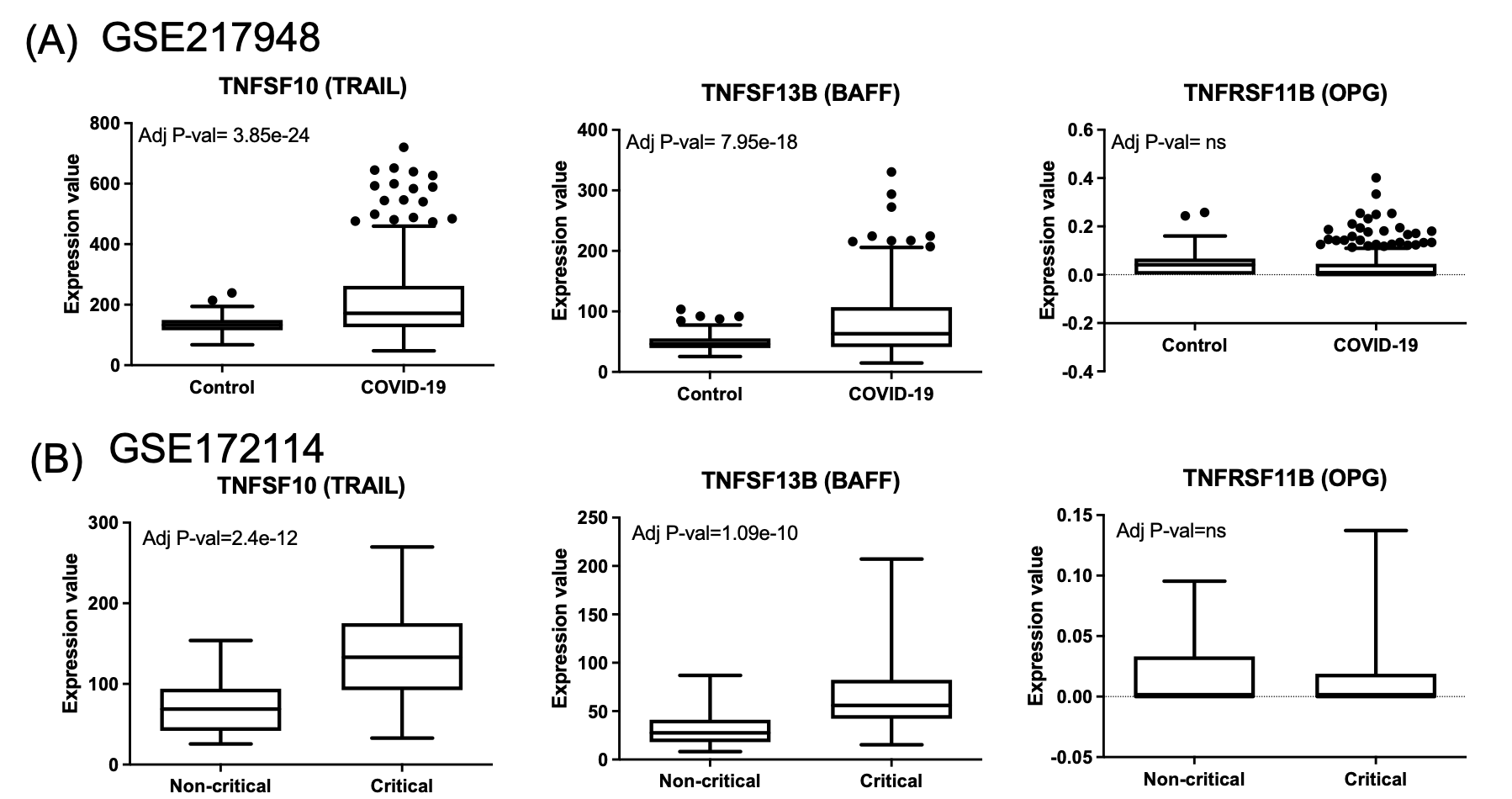


**Figure S6.** TRAIL (TNFSF10), BAFF (TNFSF13B), and OPG (TNFRSF11B) RNA transcript levels in blood samples from COVID-19 patients. Samples were grouped based on their COVID-19 status as healthy individuals (control, n=62) or COVID-19 positive patients (n=334) (A, GSE217948, PMID: 36741372), or non-critical (n=20) or critical (n=37) COVID patients (B, GSE172114, PMID: 34698500). Analysis was performed using GEO2R tool (PMID 23193258) and the Benjamini & Hochberg test for False discovery rate.
